# Primary intrahepatic malignant mesothelioma with multiple lymphadenopathies due to non-tuberculous mycobacteria: A case report and review of the literature

**DOI:** 10.3892/ol.2013.1461

**Published:** 2013-07-15

**Authors:** NORIKO INAGAKI, KAYOKO KIBATA, TAKESHI TAMAKI, TOSHIKI SHIMIZU, SHOSAKU NOMURA

**Affiliations:** First Department of Internal Medicine, Kansai Medical University, Moriguchi, Osaka 570-8506, Japan

**Keywords:** malignant mesothelioma, primary hepatic tumor, mycobacteria, literature review

## Abstract

Primary intrahepatic malignant mesothelioma (PIHMM) is an extremely rare tumor with clinicopathological characteristics that remain to be elucidated. The current study presents the case of a 68-year-old female with PIHMM and multiple lymphadenopathies due to non-tuberculous mycobacteria. The patient presented with an intrahepatic tumor, 70 mm in diameter, in the right lobe of the liver. An ultrasound-guided fine-needle aspiration biopsy of the liver tumor revealed findings that were consistent with an intrahepatic malignant mesothelioma. The systemic lymph node swellings were due to epithelioid granulomas that were caused by non-tuberculous mycobacteria. However, a hepatic rupture occurred due to the rapid growth of the liver tumor and consequently, a surgical resection was not performed. A review of the literature revealed that the clinicopathological characteristics of PIHMM are similar to those of non-occupational mesothelioma. However, PIHMM is usually a solitary tumor and is rarely associated with cavity effusion in contrast with conventional mesothelioma. Therefore, surgical resection with curative intent is often recommended for patients with PIHMM.

## Introduction

Malignant mesothelioma most commonly arises from the pleura (1), but it may also arise from the peritoneum (2), pericardium (3) and tunica vaginalis testis (4,5). However, primary intrahepatic malignant mesothelioma (PIHMM) is an extremely rare tumor (6–11). Malignant mesothelioma is known to originate from transformed mesothelial cells (12), which are not present in the hepatic parenchyma under normal physiological conditions. A possible explanation for the origin of PIHMM was proposed by Leonardou *et al*(7), who speculated that the tumor arose from mesothelial cells derived from an intruded Glisson’s capsule. However, the clinicopathological characteristics of PIHMM remain to be elucidated. The current study reports a case of PIHMM with multiple lymphadenopathies due to non-tuberculous mycobacteria and also presents the findings of a literature review. Written informed consent was obtained from the patient.

## Case report

**Figure 1:**
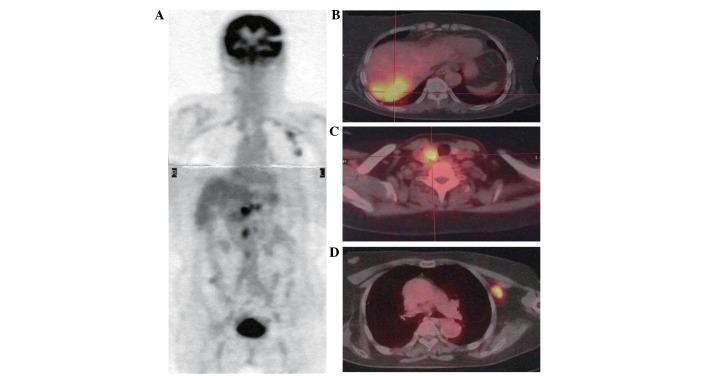
Radiological findings. (A) The fusion images of FDG-PET/CT in the coronal plane. (B) A lesion exhibiting a high FDG uptake (SUVmax, >13.5) was detected in the intrahepatic tumor of the right lobe of the liver. (C) The cervical lymph node demonstrated a high FDG uptake (SUVmax, >10.6). (D) The left axillary gland exhibited a high FDG uptake (SUVmax, >8.4). FDG-PET/CT, 2-deoxy-2-[^18^F]-fluoro-D-glucose positron emission tomography/computer tomography, SUVmax, maximum standard uptake value.

A 68-year-old female presented to Kansai Medical University Takii Hospital (Osaka, Japan) with an intrahepatic tumor and multiple lymph node swellings accompanied by a prolonged low-grade fever. The patient did not have a history of asbestos exposure or cigarette smoking. A computed tomography (CT) scan revealed cervical, axillary and abdominal para-aortic lymph node swellings, in addition to an intrahepatic tumor with a diameter of 70 mm in the right lobe of the liver. The intrahepatic tumor was heterogeneously enhanced by contrast-enhanced CT. There was no evidence of pleural effusion, ascites, pleural thickening or a peritoneal tumor. Subsequently, 2-deoxy-2[^18^F]-fluoro-D-glucose (FDG)-positron emission tomography(PET)/CT was performed, which clearly revealed a high FDG uptake in the lymph nodes and intrahepatic tumor (Fig. 1A–D). In contrast, no significant accumulation of FDG was noted in the pleura or the peritoneum. A laboratory examination showed that the C-reactive protein (CRP) and lactic dehydrogenase (LDH) levels were slightly elevated (CRP, 3.907 mg/dl; LDH, 247 U/l). However, the serum tumor marker levels were not elevated. The results of all the other laboratory examinations were within normal limits.

**Figure 2:**
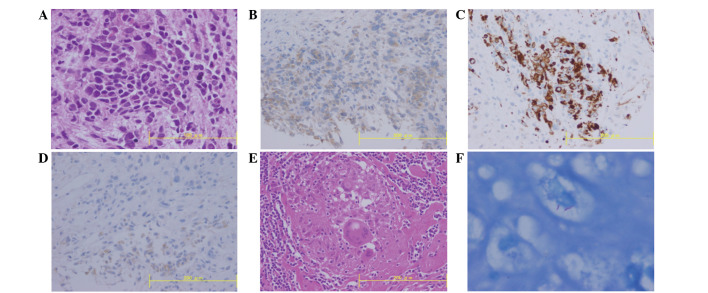
Histological findings of the biopsy specimen. (A–D) The histological findings of the biopsy specimen from the intrahepatic tumor. (A) Results of HE staining revealed the islands of polygonal tumor cells surrounded by inflammatory infiltrates (magnification, ×400). (B–D) Immunohistochemical findings of the biopsy specimen from the intrahepatic tumor. (B) The tumor cells were immunoreactive for cytoplasmic calretinin (magnification, ×200). (C) The tumor cells stained positive for Wilms tumor gene-1 (WT-1; magnification, ×200). (D) The tumor cells were immunoreactive for cytoplasmic D2–40 (magnification, ×200). (E and F) Histological findings of the biopsy specimen from the left axillary lymph node. (E) An epithelioid granuloma with multinucleated giant cells was observed in the lymph node (HE staining; magnification, ×200). (F) Ziehl-Neelsen acid-fast staining showed the presence of acid-fast bacilli (x1,000). HE, hematoxylin and eosin.

An ultrasound-guided fine-needle aspiration biopsy of the liver tumor was performed. A histological examination of the biopsy specimen revealed islands of polygonal tumor cells with a high nuclear to cytoplasmic ratio and prominent nucleoli (Fig. 2A). Few mitotic figures were noted. The tumor islands were surrounded by inflammatory infiltrates. Alcian blue and periodic acid-Schiff staining clearly demonstrated intracytoplasmic mucopolysaccharides in the tumor cells.

Immunohistochemical examination revealed that the tumor cells were negative for carcinoembryonic antigen, carbohydrate antigen 19-9, p53 and CD34. In contrast, the tumor cells stained positive for epithelial membrane protein, cytokeratin (CK) 7, CK20, CD10 and vimentin. In addition, immunohistochemical staining revealed that the tumor cells were positive for calretinin, Wilms tumor gene-1 (WT-1) and D2–40 (Fig. 2B–D). These findings strongly suggested that the intrahepatic tumor cells exhibited the phenotypical features of malignant mesothelioma.

An axillary lymph node biopsy was performed to determine whether the lymph node swellings were due to intrahepatic mesothelioma metastasis. However, the histological examination showed an epithelioid granuloma (Fig. 2E) and Ziehl-Neelsen staining revealed the presence of acid-fast bacilli (Fig. 2F). Thus, a final diagnosis of PIHMM accompanied by lymphadenopathies due to a mycobacterial infection was confirmed. PCR for *Mycobacterium tuberculosis* and the *avium-intracellulare* complex was negative. The cultivation for acid-fast bacillus failed to yield a mycobacterium species. Therefore, the pathogenic mycobacterium was not determined in this case. Since the lymphadenopathy was not due to mesothelioma metastasis, the PIHMM was considered to be a localized tumor. Therefore, a surgical resection of the liver tumor with curative intent was planned. However, a hepatic rupture occurred due to the rapid growth of the liver tumor and the general condition of the patient deteriorated. Therefore, no further investigations or treatments were possible in this case.

## Discussion

PIHMM is an extremely rare tumor. To the best of our knowledge, only six cases of PIHMM have been previously reported in the published literature (6–11). A review of these six cases, plus the present study, is summarized in Table I. The cases consisted of five male and two female patients (2.5:1), with an age range of 53–68 years (median, 62 years). Previous reviews focusing on conventional mesothelioma have shown that the male/female ratio and median age at the initial diagnosis ranged from 2.2:1–12.6:1 and 64–68 years, respectively (13–15). However, in a subgroup of non-occupational mesothelioma cases, the male/female ratio and median age at the initial diagnosis were reported to be 0.8:1–1.4:1 and 57.8–63.0 years, respectively (13,16). Thus, gender and age distribution did not differ significantly between PIHMM and non-occupational mesothelioma. Only one of the seven patients (14.3%) had a history of asbestos exposure, although it has previously been shown that conventional mesothelioma is frequently associated with asbestos exposure (58.9–86.8%) (13–15).

The prevalence of distant metastasis at the initial diagnosis has been recorded as 25.2–55.1% in conventional mesothelioma (13,16). Therefore, surgical resection is not always performed. However, all seven of the PIHMM patients reviewed in the present study had solitary tumors that were localized in the liver at the time of the initial diagnosis, and surgical resection had been performed in all cases, with the exception of the present case. All the tumors arose in the right lobe, were located in the subcapsular region and were between 3.2 and 16 cm in diameter (mean, 7.8 cm). Cavity effusion was not associated with PIHMM in any of the reviewed cases, however malignant serositis is usually observed in conventional mesothelioma (13). Three of the six patients that underwent a surgical resection relapsed post-surgery, one of which received systemic chemotherapy with pemetrexed in combination with cisplatin. Two of the three relapsed cases showed translymphatic progression. The prevalence of lymph node metastasis in conventional mesothelioma has been evaluated. Rahman *et al* reported that 18 of 53 patients (34.0%) with malignant pleural mesothelioma had positive lymph node involvement at the time of surgery (17). In addition, Edwards *et al* reported that 44 of 92 consecutive patients (47.8%) with malignant mesothelioma who underwent extrapleural pneumonectomy had positive lymph node involvement (18). Therefore, translymphatic progression is not an unusual event in malignant mesothelioma. In the present study, the survival factors were not evaluated due to the small size of the study population and inadequate survival information.

The review of the seven cases showed that five were epithelioid type (71.4%) and two were biphasic (28.6%). A sarcomatoid type was not noted among the reviewed cases. Among the conventional mesothelioma cases, the prevalence of epithelioid, biphasic and sarcomatoid subtypes was 32–67.2, 21.7–34 and 9.8–33%, respectively (13–16). The distribution of the histological types among the non-occupational mesotheliomas was not dissimilar to that among the conventional mesotheliomas (epithelioid, 64.9%; biphasic, 15.3% and sarcomatoid, 6.1%) (19). Therefore, the distribution of the histological subtypes in PIHMM was equivalent to that in conventional mesothelioma. The tumor phenotypes of the reviewed cases are summarized in Table II. Previously, several studies had been conducted to clarify the phenotypical features of malignant mesothelioma using immunohistochemistry and these results are also listed in Table II. Consequently, this literature review clearly showed that PIHMM and conventional mesothelioma do not differ significantly with respect to cellular phenotype (20–28).

In the present case, multiple lymphadenopathies were observed in addition to the liver tumor. FDG-PET/CT did not demonstrate the difference in metabolic behavior between the intrahepatic tumor and lymphadenopathies. However, the lymph node lesions were identified to be non-cancerous granulomas due to the presence of mycobacteria. FDG-PET/CT examination was insufficient to differentiate epithelioid granuloma due to mycobacterial infection from malignant mesothelioma in this case. Studies have also demonstrated that mycobacteriosis commonly causes increased ^18^F-FDG uptake (29–32). Thus, an aggressive biopsy is warranted when multiple lymph node swellings are associated with a malignant tumor.

The present study describes a case of PIHMM with multiple lymphadenopathies due to non-tuberculous mycobacteria. A literature review clearly indicated that the clinicopathological characteristics of PIHMM are similar to those of non-occupational mesothelioma. However, the tumors in all the reviewed cases were solitary tumors that were localized in the liver and none were accompanied by cavity effusion. Further investigation of the pathophysiological features of PIHMM is required to develop an appropriate treatment strategy.

## Figures and Tables

**Table I:** Characteristics of patients with PIHMM.

First author, year (ref.)	Age, years	Gender	Asbestos exposure	Histology	OS, months	Location (segment)	Size, cm	Treatment	Relapse
Imura *et al,* 2002 (6)	64	M	(−)	Ep	40	Rt (S7)	3.2	Surg	None
Leonardou *et al*, 2003 (7)	54	F	N/E	Ep	2	Rt	16.0	Surg	None
Gütgement *et al*, 2006 (8)	62	M	(−)	Ep	5	Rt	5.8	Surg	LNR
Kim *et al*, 2008 (9)	53	M	(−)	Bp	N/E	Rt	13.0	Surg	DI
Sasaki *et al*, 2009 (10)	66	M	(+)	Bp	6	Rt (S8)	4.4	Surg	None
Buchholz *et al*, 2009 (11)	62	M	(−)	Ep	36	Rt (S5, S8)	5.8	Surg	LNR
Present case	68	F	(−)	Ep	3	Rt (S7)	7.0	BSC	N/E

PIHMM, primary intrahepatic malignant mesothelioma; OS, overall survival time; M, male; F, female; N/E, not evaluated; Ep, epithelioid; Bp, biphasic; Surg, surgical resection; BSC, best supportive care; Rt, right lobe; LNR, translymphatic relapse; DI, direct invasion.

**Table II:** Immunohistochemical phenotypes of PIHMM and conventional mesothelioma.

Items	D2–40	WT-1	Calretinin	Vimentin	p53	CK7	CK20
PIHMM, degree of staining							
Imura *et al*, 2002 (6)	N/E	N/E	(+)	N/E	(+)	N/E	N/E
Leonardou *et al*, 2003 (7)	N/E	N/E	(+)	(+)	N/E	N/E	N/E
Gütgement *et al*, 2006 (8)	(+)	(+)	(+)	(±)	(+)	N/E	(−)
Kim *et al*, 2008 (9)	N/E	N/E	(+)	N/E	N/E	(+)	(−)
Sasaki *et al*, 2009 (10)	(+)	(+)	(+)	(+)	(+)	(+)	N/E
Buchholz *et al*, 2009 (11)	(+)	(+)	(+)	(±)	(+)	N/E	(−)
Present case	(±)	(+)	(+)	(+)	(−)	(+)	(+)
Conventional mesothelioma^a^, % (refs.)	85 (20)	55–100 (20,21,23)	39.8–100 (20–22)	27.7–96 (21,22,24)	45–69.6 (22,25)	65–100 (26–28)	0 (26–28)

a The proportion of positive staining (refs. 20–28).

PIHMM, primary intrahepatic malignant mesothelioma; N/E, not evaluated; WT-1, Wilms tumor gene-1; CK, cytokeratin; (+), positive staining; (−), negative staining; (±), weakly positive staining.
